# The Staff Observation Aggression Scale – Revised (SOAS-R) – adjustment and validation for emergency primary health care

**DOI:** 10.1186/s12913-018-3157-z

**Published:** 2018-05-08

**Authors:** Tone Morken, Valborg Baste, Grethe E. Johnsen, Knut Rypdal, Tom Palmstierna, Ingrid Hjulstad Johansen

**Affiliations:** 1 National Centre for Emergency Primary Health Care, Uni Research Health, Kalfarveien 31, 5018 Bergen, Norway; 2 Uni Research Health, Bergen, Norway; 3 Centre for Research and Education in Forensic Psychiatry, Haukeland University Hospital, Bergen, Norway; 4 Centre for Psychiatry Research, Department of Clinical Neuroscience, Karolinska Institutet, Stockholm, Sweden

**Keywords:** Aggression, Workplace violence, Primary health care

## Abstract

**Background:**

Many emergency primary health care workers experience aggressive behaviour from patients or visitors. Simple incident-reporting procedures exist for inpatient, psychiatric care, but a similar and simple incident-report for other health care settings is lacking. The aim was to adjust a pre-existing form for reporting aggressive incidents in a psychiatric inpatient setting to the emergency primary health care settings. We also wanted to assess the validity of the severity scores in emergency primary health care.

**Methods:**

The Staff Observation Scale - Revised (SOAS-R) was adjusted to create a pilot version of the Staff Observation Scale – Revised Emergency (SOAS-RE). A Visual Analogue Scale (VAS) was added to the form to judge the severity of the incident. Data for validation of the pilot version of SOAS-RE were collected from ten casualty clinics in Norway during 12 months. Variance analysis was used to test gender and age differences. Linear regression analysis was performed to evaluate the relative impact that each of the five SOAS-RE columns had on the VAS score. The association between SOAS-RE severity score and VAS severity score was calculated by the Pearson correlation coefficient.

**Results:**

The SOAS-R was adjusted to emergency primary health care, refined and called The Staff Observation Aggression Scale - Revised Emergency (SOAS-RE). A total of 350 SOAS-RE forms were collected from the casualty clinics, but due to missing data, 291 forms were included in the analysis. SOAS-RE scores ranged from 1 to 22. The mean total severity score of SOAS-RE was 10.0 (standard deviation (SD) =4.1) and the mean VAS score was 45.4 (SD = 26.7). We found a significant correlation of 0.45 between the SOAS-RE total severity scores and the VAS severity ratings. The linear regression analysis showed that individually each of the categories, which described the incident, had a low impact on the VAS score.

**Conclusions:**

The SOAS-RE seems to be a useful instrument for research, incident-recording and management of incidents in emergency primary care. The moderate correlation between SOAS-RE severity score and the VAS severity score shows that application of both the severity ratings is valuable to follow-up of workers affected by workplace violence.

**Electronic supplementary material:**

The online version of this article (10.1186/s12913-018-3157-z) contains supplementary material, which is available to authorized users.

## Background

A substantial number of workers in emergency primary health care experience aggressive behaviour such as verbal aggression, threats or physical violence from patients or visitors [1]. These incidents are a threat to the safety and wellbeing of both health care workers and patients [2, 3] A review on workplace violence states that adopting simple incident-reporting procedures is helpful in ensuring safe working environments [4]. Such procedures can protect the worker from retribution, ensure managerial support, and support the implementation of preventive solutions. Simple incident-reporting procedures exist for inpatient, psychiatric care [5]. However, to our knowledge no similar form for simple incident-reporting has been developed for other health care settings. Health care services providing emergency and accident medical care are services at high risk of workplace violence [6–8]. These services differ from inpatient, psychiatric care by providing easily accessible and unscheduled care for an unselected and largely unfamiliar patient population. They deal with the whole range of clinical scenarios, from minor medical illnesses to serious medical conditions, traumas, toxic exposures, substance abuse and psychiatric emergencies.

In Norway, emergency and accident care is provided by general practitioners in emergency primary care clinics, and we know that one in three employees has been physically attacked during their worktime career [1]. Aggressive incidents seem to be underreported [9], and simple incident-reporting may increase knowledge about and ability to deal with workplace violence. However, the forms developed for inpatient settings have limited relevance outside hospital wards due to differences in service organization, range of clinical scenarios and familiarity with the patients. The aim of this study was therefore to adjust a pre-existing form for reporting aggressive incidents in a psychiatric inpatient setting to the emergency primary health care settings. We also wanted to assess the validity of the severity scores in emergency primary health care.

## Methods

The incident reporting form used in this study was developed from the Staff Observation Scale - Revised (SOAS-R). SOAS-R is easy and quick to fill in, can be used without previous training, and has been shown to give a valid approximation of the severity of aggressive incidents [5, 10]. In 1987, Palmstierna and Wistedt constructed the Staff Observation Scale to monitor frequency, nature and severity of aggressive incidents which are damaging or threatening to objects or humans in psychiatric wards [11]. The scale was revised in 1999, forming the currently used SOAS-R [5], and is used in psychiatric settings worldwide [12]. The SOAS-R report form is completed when a staff member observes aggressive behaviour on the part of a patient. It consists of five columns (categories), and each column comprises several options to characterise the actual incident. Several options can be marked in each column. The five columns have the following themes: 1) The provocation that leads to the aggressive incident; 2) The means used by the aggressor during the incident; 3) The target of aggression; 4) The consequences for victims; and 5) The immediate measures taken to stop or control aggressive behaviour.

In the original SOAS, the severity scores were developed based on face-validity [11], while in SOAS-R the scoring system was further developed and designed statistically to increase the validity [5, 13]. A total score system was developed to rate overall severity of an aggressive incident [5]. Each option in the columns was given a score, and the column score equalled the highest scoring option of each column. The total severity score was derived by adding the five column scores. The SOAS-R total severity score ranges from 0 to 22 points, with scores of 0–7 indicating mild, 8–15 moderate, and 16–22 severe severity [12]. A SOAS-R severity score of 9 or more includes all incidents where physical pain or injury is inflicted. A score of 9 or more also includes all physical attacks causing fear of harm to the victim, as well as attacks with dangerous objects directed at a person [14].

In addition to the five columns, a 100-mm Visual Analogue Scale (VAS) is often added to the SOAS-R form to judge the severity of the incident [5]. The VAS is found to be suitable for assessment of subjective phenomena [15], and has also been validated for assessing occupational stress [16]. On the VAS, the worker marked the severity of the aggression on a scale ranging from “not severe at all” (at the 0-end of VAS) to “extremely severe” (at the 100-end of the VAS).

### Setting

Emergency primary care in Norway is organized as casualty clinics or as part of a regular general practitioner’s surgery. Many of the units are small, isolated and geographically distant from the hospital. The number of staff on duty varies from one to several persons, mainly physicians (mandatory) and nurses. The physicians primarily see patients at the clinic, but they also conduct home visits and participate on site in emergencies outside hospitals. The clinics provide walk-in services, and as a matter of policy, they are easily accessible to the public. They give care to all persons in need who reside within a defined geographical area. The clinics handle all types of medical emergencies, and are gatekeepers for all kinds of secondary medical and psychiatric care. Most patients are treated at the clinic without further referral to secondary care.

### Development of the pilot version of the SOAS-RE

A number of adjustments were made to adapt the SOAS-R to the setting of emergency primary health care (SOAS-RE). The changes were based on input from nurses and doctors working in emergency primary health care. Background questions included location for the incident (clinic, phone, home visit/emergency call out); the worker’s age, occupation, gender, whether the worker was alone on duty; and information about the aggressor’s gender, mental health and present drug or alcohol use or presence of influences from these.

New options were added in each of the existing columns and an additional column was added based on findings from qualitative studies on factors influencing workplace violence in emergency primary health care [17, 18]. In column 1 (Provocation of aggressive behaviour), the new options “the person had to wait”, “the person disagreed about assessment/advice” and “involuntary assessment of health condition” were added. In column 2 (Means used by the aggressor), “used/had weapon” and “used/ had pointed weapon” were added. In column 3 (Target of aggression), “physician”, “nurse”, “ambulance personnel”, “security guard”, and “police” were added. In column 4 (Consequences for victim) “psychological/emotional stress”, and “needs to be taken off duty” were added. In column 5 (Measures to stop aggression) “withdrew from situation/ended call”, “complied with the person’s wish”, and “asked the person to leave the site” were added. The new and sixth column was used for information about persons involved to stop the aggression. This information was judged as valuable to complete the description of the aggressive episode, but was not included in the severity scoring system. Contrary to the traditional psychiatric inpatient setting where only nursing staff is involved in handling an incident, the emergency primary health care setting includes different persons or occupations, like physicians, nurses, ambulance personnel, security guards, police, other patients and next-of-kin.

The scores ranged from 0 to 2 (first column (Provocation)), 0 to 3 (second column (Means used)), 0 to 4 (third column (Target of aggression), 0 to 9 (fourth column (Consequence(s) for victim)). All SOAS-RE severity scores were assigned based on the scores used in SOAS-R and an adjustment of these after a discussion in the project group about the relative severity of each item. In the fourth column, the new option “psychological/emotional stress” was assigned the SOAS-RE severity score 4. As damaged objects were considered less severe than psychological/emotional stress, the existing item “objects damaged” was assigned the SOAS-RE severity score 2, as opposed to severity score 4 in SOAS-R. The scores of the fifth column (Measure(s) to stop aggression) ranged from 0 to 4. All items in column 6 had a severity score of 0 and were therefore not added to the SOAS-RE severity score but used only for factual information about the incident. Thus, the possible range of the SOAS-RE total severity score was 0–22 points.

### Sample

In validation studies, it is recommended that more than 100 recordings be included [19]. Based on one per thousand contacts with threatening behaviour in casualty clinics [20] and about 300 contacts per thousand inhabitants per year [21], we estimated that the casualty clinics recruited to document incidents had to cover more than 300,000 inhabitants. Information about the study was given at emergency primary health care conferences. Eleven emergency primary care clinics self-recruited to the study, but one of the clinics did not send in any forms during the study period. The remaining ten clinics were geographically spread throughout Norway and covered a total population of 1.3 million inhabitants. The observation period was 12 months (2016).

### Procedure

Before the data collection started, a sub-investigator visited all the casualty clinics and gave verbal and written instructions to the contact person at the clinic on how to conduct the collection of data. The contact person was given the following instruction: “After an aggressive incident, the health worker involved in the aggressive situation should complete the SOAS-RE form. Then, the severity of the aggressive incident should be assessed by VAS scale ranging from “not severe at all” (left end, 0 mm) to “extremely severe” (right end, 100 mm). The contact person collects the anonymously completed forms and forwards them to the National Centre for Emergency Primary Health Care.” The contact person was given the responsibility for informing the colleagues and making sure that the form was used after incidents. The sub-investigator had email communication with the contact persons each month to remind them about the data collection and to confirm whether there had been any incidents the previous month.

After receiving the form, the researchers calculated a total severity score based on predefined scores for each item. Neither the contact person nor the worker received information about how the SOAS-RE severity scores were calculated.

### Statistical analyses

Background characteristics are given as mean, standard deviation (SD) and percentages. Mean SOAS-RE and VAS severity scores, SD and 95% confidence interval (CI) are presented for each column and for each group of items holding the same SOAS-RE value. Variance analyses were used to test gender and age differences of the victims in SOAS-RE severity score and in VAS severity score. Linear regression analysis was performed to evaluate the relative impact each of the five SOAS-RE columns had on the VAS score. The association between SOAS-RE severity score and VAS severity score was calculated by the Pearson correlation coefficient. Items that were associated with higher VAS scores were identified, and the severity scoring system could eventually be improved.

## Results

A total of 350 SOAS-RE forms from ten different casualty clinics were registered of which 26 forms lacking VAS severity scores and 33 forms lacking SOAS severity scores were excluded from the analysis. The remaining 291 forms were completed by 202 female workers (69.4%) and 76 males (26.1%). Information on gender was missing in 13 forms (4.4%). There were 231 nurses (79.4%), 41 physicians (14.1%), 13 other professionals (4.5%) and 6 with unspecified profession (2.1%). Four persons (1.4%) reported that they had been alone on duty.

**Fig. 1:**
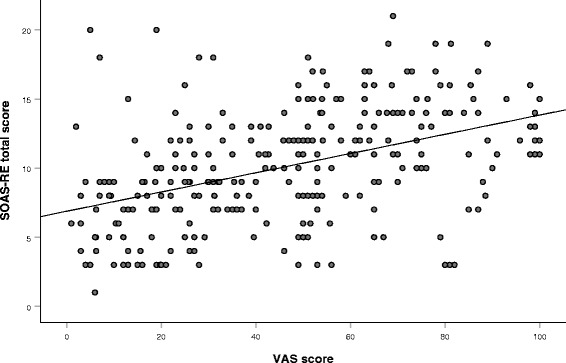
Scatterplot of the relationship between SOAS-RE and VAS severity scores (*n* = 291)

SOAS-RE total severity scores ranged from 1 to 22. The mean severity score of SOAS-RE was 10.0 (SD = 4.1) and the mean VAS severity score 45.4 (SD = 26.7). The scatterplot (Fig. 1) shows the relationship between SOAS-RE and VAS severity scores. The Pearson correlation between the two scores was 0.45 (*p* < 0.001). There were two outliers defined as standardized residual greater than 3. When these were omitted, the correlation was 0.48 (*p* < 0.001).

No gender differences were found in SOAS-RE severity score (*p* = 0.145) or in the VAS severity scores (*p* = 0.071). There was no association between the age of the worker and the SOAS-RE severity score (*p* = 0.950) or the VAS severity score (*p* = 0.638).

### Validation of the severity scores of SOAS-RE

Table 1 shows the mean SOAS-RE severity rating and mean VAS scores for the equally rated items in each of the five columns. In general, there was an increase in mean severity rating and a corresponding increase in mean VAS scores. However, in column 1 (Provocation) the item “involuntary assessment” had a lower mean VAS severity score than expected, and the cases with “no understandable provocation” had a higher mean VAS severity score than the cases with “involuntary assessment”. Similarly, the cases with “nothing/nobody” in column 3 (Target of aggression) had by far the higher mean VAS severity scores than when the aggression had a defined target. These five “nothing/nobody” cases had a wide 95% CI (37.6–92.2), and included two incidents where the patient was under the influence of drugs or alcohol and had a syringe-needle containing blood, two cases of threats using hand and foot, and one case of verbal aggression. In column 4, the item “object(s) damaged” had a lower mean VAS score than expected. However, this item included only two cases.

**Table 1:** Mean VAS Scores, standard deviations, confidence interval, mean SOAS total score by max score in each column and SOAS-RE severity points assigned (*n* = 291)

			VAS^a^ severity score				
SOAS-RE column	Categories	n	Mean	SD	95% CI	Mean SOAS-RE total score^b^	SOAS-RE points assigned^c^
1. Provocation	Had to wait/ was denied something/disagrees about advice	191	43.1	26.8	39.3–46.8	9.5	0
	No understandable provocation	78	50.9	26.6	45.2–57.4	10.4	1
	Involuntary assessment	22	45.8	24.3	36.2–55.8	13.8	2
2. Means used by aggressor	Verbal aggression	98	30.9	20.4	26.0–35.2	7.3	0
	Threats/ordinary objects	79	46.5	25.7	40.6–52.4	9.7	1
	Parts of the body (hand, foot, other)	99	55.3	26.9	49.9–60.7	12.4	2
	Dangerous objects or methods	15	69.6	18.7	59.5–78.3	14.1	3
3. Target of aggression/victim	Nothing/nobody	5	71.2	37.2	37.6–92.2	9.0	0
	Objects	6	46.9	17.2	34.3–59.5	9.2	1
	Staff member/security/police	247	44.3	26.2	41.1–47.6	9.9	3
	Other patients/persons	33	49.5	29.3	39.3–58.9	11.3	4
4. Consequence(s) for victim(s)	None	77	32.9	25.0	27.6–39.1	5.4	0
	Object(s) damaged	2	50.5	2.1	49.0–52.0	8.0	2
	Psychological stress	74	40.1	25.1	34.4–45.9	9.0	4
	Felt threatened	109	55.2	24.5	50.5–60.0	12.3	6
	Physical consequences (pain, injury, need for treatment, taken off duty)	29	55.4	27.5	45.4–65.5	16.7	9
5. Measure(s) to stop aggression	None/talk with patient/took the person aside/complied with the person’s wish	158	38.5	24.9	34.6–42.6	7.8	0
	Asked the person to leave/medication	74	50.1	25.0	44.7–56.0	11.5	2
	Held with force/forced to leave	59	58.1	27.8	51.2–64.7	14.2	4

^a^*VAS* visual analogue scale, *SOAS-RE* Staff Observation Aggression Scale -revised emergency, *SD* Standard deviation

^b^Mean SOAS-RE total score gives the mean total sum scores for all incident reports where this alternative had been reported

^c^SOAS-RE points assigned shows the severity score given to each alternative within the individual column

The linear regression analysis showed that each of the columns had a low impact on the VAS score (Table 2). ‘Means used by aggressor’, ‘Consequence(s) for victim(s)’ and ‘Measure(s) to stop aggression’ had significant impact, but explained only 1.8 to 10.4% of the variation. Means used by aggressor was the strongest predictor. The total model explained 25.6% of the variance in VAS scores.

**Table 2:** Standardized Beta coefficients and explained variance for VAS score estimated from SOAS-RE column score by a multiple linear regression (*n* = 291)

SOAS-RE^a^ column	Standardized Beta	Explained variance	*p*
2. Means used by aggressor	0.33	0.105	< 0.001
4. Consequence(s) for victim(s)	0.23	0.051	< 0.001
5. Measure(s) to stop aggression	0.13	0.016	0.026
3. Target of aggression	−0.03	0.001	0.524
1. Provocation	0.007	< 0.001	0.895

^a^*SOAS-RE* Staff Observation Aggression Scale -revised emergency

### Adjustment of the pilot version of SOAS-RE

Based on the experience with the pilot version of SOAS-RE, the form was refined. In the background information, we changed the question “Were you alone on duty?” to “Were you alone in the situation?”. We expected that this change of question would yield future data that will provide valid information about being alone in the situation with the aggressor, although more staff might be present at the clinic. In column 2, “means used by the aggressor”, the item “other dangerous objects” was changed to “other dangerous items including syringe” based on the finding that some forms had a free-text description of use of syringe without having marked the box “other dangerous objects”. Additional file 1 shows the final version in English.

## Discussion

Several measures for incident reporting of patient violence and aggression towards healthcare providers have been developed, most of them for hospital settings [22]. The SOAS-R was adjusted to emergency primary health care, and found to be well suited for this context, as it is short, easy to fill in, and combines the visual analogue severity scale with a description of elements of the actual incident. Furthermore, as opposed to similar registration forms like Arnetz Violence Incident Form [23], the SOAS-RE helps the healthcare leaders and risk managers to identify and act on the severity of the incident itself and by judging the subjective impact the incident had on the healthcare worker involved, shown by the healthcare worker’s VAS score. The new form can thus be used both to register incidents and to identify employees in need to follow up independent of the severity of the actual incident reported. The new form was named The Staff Observation Aggression Scale - Revised Emergency (SOAS-RE). The mean severity scores of 10.0 in SOAS-RE were similar to SOAS-R mean severity scores found in other studies, ranging from 9.2 to 11.0 [12]. There was a significant correlation of 0.45 between the VAS severity ratings and the SOAS-RE severity scores. However, the correlation was higher between VAS and the original SOAS-R (0.62) [5]. The moderate relationship between VAS and SOAS-RE severity scores suggest that SOAS-RE and VAS cannot be substituted for one another in measuring severity of aggressive incidents. As has been claimed by the developer of the SOAS, the subjective measurement by VAS severity score may be more influenced by intraobserver variation than the established severity scoring system of SOAS-R [5]. The staff members’ individual emotional reactions, previous experiences and attributions could affect their propensity to report aggressive episodes and perceived subjective severity [24]. The VAS severity score could therefore be used to identify individuals who perceive the incident as severe, and to ensure follow up of vulnerable individuals independently of the SOAS-RE score.

The SOAS-RE ratings are part of a comprehensive system for monitoring aggressive behaviour and provide a broad array of characteristics of aggressive incidents as well as their severity [10]. The SOAS-RE form may be suitable primarily to describe the content of the incidents and to prioritize preventive measures based on the factual information.

Column 2 (Means used by aggressor) was most strongly connected to the VAS severity score in SOAS-RE and were more strongly connected to the VAS than was found in SOAS-R [5]. It is likely that the intrusiveness of the aggression is more powerful in determining the emotional response, than other aspects of the incident. This might be the case particularly in a setting where aggression is mostly unexpected and the health care worker is unfamiliar with the aggressor. The other columns reflected the same relative contribution as in SOAS-R.

In column 1, involuntary assessment of health condition as provocation of aggressive behaviour had a relatively low mean VAS score, while the SOAS-RE score was relatively high. The professionals judged involuntary assessment as of the highest severity in column 1 in the SOAS-RE scoring system. However, the staff may expect violence in situations with involuntary assessment [18], and might therefore mobilize other personnel or implement other preventive measures to increase their own safety. The situation might therefore be perceived as safer than when violence is completely unexpected, as it might be more difficult to receive the help needed in the latter situation.

### Study strengths and limitations

The strength of this study is the prospective design, and that the collection of data was standardized. The data was collected from casualty clinics from various parts of Norway and with different organizational assets. The data therefore describe a broad variation in incidents and severity, and the description of incidents may be generalizable to emergency primary health care settings. However, there might be underreporting of incidents, especially less severe cases, as has been shown in previous studies of SOAS-R [12]. We therefore have little information about the validity of SOAS-RE in less severe incidents. In addition, some of the items in SOAS-RE had very low incidence numbers, and we were therefore unable to assess the validity of those scores.

### Further research

Validating a tool is not sufficient for practical use. For successful implementation, the tool also needs to be easy, user-friendly and the incident reporting needs to be positively supported by the managers [25]. The usefulness of the tool has to be further evaluated after implementation in casualty clinics.

## Conclusions

The SOAS-RE seems to be a useful instrument for research, incident-recording and management of incidents in emergency primary care. The moderate correlation between SOAS-RE severity score and the VAS severity score shows that application of both these severity ratings is needed to ensure appropriate follow up of workers affected by workplace violence.

## Additional file

Additional file 1: Appendix_SOAS-RE: English version of SOAS-RE. (PDF 80 kb)

## Abbreviations

CI: Confidence interval
SD: Standard deviation
SOAS-R: The staff observation aggression scale – revised
SOAS-RE: The staff observation scale – revised emergency (SOAS-RE).
VAS: Visual analogue scale
