# Supplementary material for: The Staff Observation Aggression Scale – Revised (SOAS-R) – adjustment and validation for emergency primary health care
Source: BMC Health Serv Res. 2018 May 8;18:335. doi: 10.1186/s12913-018-3157-z (PMC5941337; doi:10.1186/s12913-018-3157-z)
Supplement: Supplementary file 1 — Appendix_SOAS-RE: English version of SOAS-RE. (PDF 80 kb) [file 12913_2018_3157_MOESM1_ESM.pdf]

SOAS-RE

The Staff Observation Aggression Scale – Revised – Emergency primary care

The form is to be completed by the staff member who has exposed to aggressive behavior defined as: any verbal, nonverbal or physical behavior that was threatening (to self, others, property), or physical behavior that did harm (to self, others, property).

1. When was the incident?

   Date (dd/mm 20yy)

   .... / .... 20 ....

   Time ....

2. Where was the incident?

   At the clinic ☐

   Over the phone ☐

   At home visit ☐

   Other..

3. **Staff member exposed to incident:**

   Age ....

   Occupation ....

   Sex: Female ☐ Male ☐

4. Were you alone in the situation?

   Yes ☐ No ☐

5. **Aggressor:**

   Who: Patient ☐ Next-of-kin ☐ Other ....

   Sex: Female ☐ Male ☐

6. Does the aggressor have a known mental illness?

   Yes ☐ No ☐ Unknown ☐

7. Was the aggressor under influence of drugs or alcohol?

   Yes ☐ No ☐ Unknown ☐

Mark at least one alternative in each of the six columns below

Provocation of aggressive behaviour

- No understandable provocation ☐

Provoked by:

- Person had to wait ☐
- The person was denied something ☐
- The person disagreed about assessment/ advice ☐
- Involuntary assessment of health condition ☐
- Other, describe: ☐

....

Means used by the aggressor

- Verbal aggression ☐
- Threat ☐

Ordinary objects

- Room contents/ objects ☐

Parts of the body

- Hand ☐
- Foot ☐
- Other, describe: ☐

....

Dangerous objects or methods

- Attempt of strangulation ☐
- Used/had weapon ☐
- Used/had pointed weapon ☐
- Other dangerous objects incl. syringe ☐

Target of aggression

- None ☐
- Furniture/objects ☐
- Physician ☐
- Nurse ☐
- Ambulance personnel ☐
- Security guard ☐
- Police ☐
- Other patients ☐
- Other persons, describe: ☐

....

Consequence(s) for victim(s)

- None ☐

Objects

- Damaged ☐

Person

- Psychological/ emotional stress ☐
- Felt threatened ☐
- Pain ☐
- Visible injury ☐
- Need for treatment by a physician ☐
- Needed to be taken off duty ☐
- Other, describe: ☐

....

Measure(s) to stop aggression

- None ☐
- Talked to person ☐
- Took the person aside ☐
- Withdrew from situation/ ended call ☐
- Complied with the person's wish ☐
- Asked the person to leave the site ☐
- Forced the person to leave ☐
- Held the person by force ☐
- Medication ☐
- Other, describe: ☐

....

Persons involved in measure(s) to stop aggression

- Physician ☐
- Nurse ☐
- Ambulance personnel ☐
- Security guard ☐
- Police ☐
- Other patients ☐
- Next-of-kin ☐
- Others, describe: ☐

....

Adjusted from "The Staff Observation Aggression Scale–Revised (SOAS-R)" (Nijman et.al. 1999)

Visual Analogue Scale (VAS)

Mark on the line

The aggressive incident was

|——————————————————————|

not severe at all — extremely severe
